# Innate lymphoid cells are double‐edged swords under the mucosal barrier

**DOI:** 10.1111/jcmm.16856

**Published:** 2021-08-10

**Authors:** Zhen Duan, Mandie Liu, Lin Yuan, Xizi Du, Mengping Wu, Yu Yang, Leyuan Wang, Kai Zhou, Ming Yang, Yizhou Zou, Yang Xiang, Xiangping Qu, Huijun Liu, Xiaoqun Qin, Chi Liu

**Affiliations:** ^1^ Department of Physiology School of Basic Medicine Science Central South University Changsha China; ^2^ Centre for Asthma and Respiratory Disease School of Biomedical Sciences and Pharmacy Faculty of Health and Medicine University of Newcastle and Hunter Medical Research Institute Callaghan NSW Australia; ^3^ Department of Immunology School of Basic Medicine Science Central South University Changsha China; ^4^ China‐Africa Infectious Diseases Research Center Xiangya School of Medicine Central South University Changsha China

**Keywords:** epithelial cells, innate lymphoid cells (ILCs), mucosal barrier, mucosal diseases

## Abstract

As the direct contacting site for pathogens and allergens, the mucosal barrier plays a vital role in the lungs and intestines. Innate lymphoid cells (ILCs) are particularly resident in the mucosal barrier and participate in several pathophysiological processes, such as maintaining or disrupting barrier integrity, preventing various pathogenic invasions. In the pulmonary mucosae, ILCs sometimes aggravate inflammation and mucus hypersecretion but restore airway epithelial integrity and maintain lung tissue homeostasis at other times. In the intestinal mucosae, ILCs can increase epithelial permeability, leading to severe intestinal inflammation on the one hand, and assist mucosal barrier in resisting bacterial invasion on the other hand. In this review, we will illustrate the positive and negative roles of ILCs in mucosal barrier immunity.

## 1 INTRODUCTION

The mucosal barrier is the first contact surface with the external environment, which forms a powerful shield to fight against pathogens. Many studies indicate that mucosal barrier injury underlies pathogenesis of many mucosal diseases. Of note, in recent years, accumulating evidence has shown that mucosal‐resident innate lymphoid cells (ILCs) are essential in mucosal diseases. ILCs can respond to damaged mucosal‐derived ‘risk signals’ sensitively and quickly due to the close proximity of the damaged site, triggering ILC‐mediated subsequent inflammatory response and tissue repair. Significant attention is paid to the role of ILCs that may also be of great therapeutic potential in mucosal diseases. In this review, we focus on the physiological and pathological functions of ILCs in the mucosal barrier. Meanwhile, we summarize the positive and negative roles of ILCs in mucosal barrier immunity. When external irritations stimulate mucosae, ILCs play a positive role to maintain mucosal homeostasis by alleviating inflammation and promoting tissue repair. However, excessive ILCs can also play a negative role by recruiting inflammatory cells and pro‐inflammatory cytokines, leading to damage the integrity of mucosae and accelerate the pathological process of chronic mucosal diseases.

## 2 MUCOSAL BARRIER ACTS MORE THAN A PHYSICAL BARRIER

The mucosal barrier is the first site to contact with the environmental antigens and pathogens, such as viruses, fungi, parasites and bacteria. The intact epithelia, cilia and mucus together constitute a strong physical barrier against external stimulus. A variety of allergens and stimuli, except probiotics, are detrimental to the host. After prolonged and/or extensive exposure to ozone, house dust mite (HDM), bacteria and viruses, etc., epithelial structural proteins such as E‐cadherin and zonula occludens‐1 (ZO‐1) were destructed, resulting in decreased epithelial tightness and barrier damage.^1^, ^2^, ^3^, ^4^ The dysfunction of epithelia is a common pathological characteristic of mucosal diseases. Various degrees of epithelial damage are the key determinant of mucosal diseases such as asthma, chronic obstructive pulmonary disease (COPD) and inflammatory bowel diseases (IBD) and the degree of epithelial damage is positively correlated with the severity of diseases.^5^, ^6^, ^7^ Once the mucosal system is too vulnerable to identify and/or eliminate noxious stimuli in time, pathogen‐induced inflammation may damage the mucosal barrier excessively, leading to severe infection, cytokine storm or even death eventually.^8^, ^9^, ^10^, ^11^

The mucosal system can be involved in many mucosal diseases in addition to its physical barrier function. More importantly, it also has a powerful immune function. When the structure and function of the mucosal barrier are damaged, mucosal epithelia would secret cytokines and inflammatory mediators to trigger systemic immune response and eliminate pathogens, which in turn restore the homeostasis of the mucosal barrier.^12^, ^13^ In the process of mucosal immune response, ILCs have attracted a substantial amount of attention due to special distribution and function. A large number of studies have demonstrated that the activation and differentiation of ILCs have great biological significance in regulating local inflammation and promoting tissue repair in the mucosal barrier. The distribution and function of ILCs in the mucosal barrier and the involvement of ILCs in mucosal diseases deserve further study.

## 3 PROFILE OF ILCS

In recent years, ILCs have aroused broad attention in the mucosal immunity and the homeostasis of mucosal barrier. ILCs are renewed from progenitors, and recent studies have identified that CD117^+^ ILC precursors derived from CD34^+^ hematopoietic stem cells can generate almost all mature ILC subsets (except for interleukin‐17A^+^ ILC3s) under appropriate environmental signals.^14^, ^15^ Interestingly, CD117^+^ ILC precursors can circulate in the peripheral blood or reside in the tissues, but lineage‐specified progenitors of ILC3s can be detected in tonsil and intestinal lamina propria but not in peripheral blood.^16^ Of note, ILCs are sentinel cells distributed widely in the mucosal barrier, while most mature ILCs are resident in intestine lamina propria, lung and liver.^17^ As ILCs are similar to T cells in terms of transcription and function, there are ILC subsets such as ILC1, ILC2, ILC3 and ILCreg by reference to the subsets of T cells (Table 1).^18^, ^19^, ^20^

**TABLE 1:** Features of innate lymphoid cell (ILC) subsets

Nomenclature	Lineage‐defining transcription factors	Stimulation	Effector molecules
Group 1 ILCs (ILC1)	T‐bet	IL−12, IL−15, IL−18	IFN‐γ, TNF‐α
Group 2 ILCs (ILC2)	GATA3	IL−25, IL−33, TSLP	IL−4, IL−5, IL−9, IL−13
ILC2_(10)_	GATA3	IL−33, papain	IL−10
Group 3 ILCs (ILC3)	RORγt	IL−1, IL−23	IL−17, IL−22, IFN‐γ, GM‐CSF
Regulatory ILCs (ILCreg)			
ILCreg (lung)	GATA3	RA, IL−2, IL−13	IL−10
ILCreg (intestine)	Id3, Sox4	TGF‐β	IL−10

ILCs are developed and expanded within peripheral organs, and then acute infections or tissue damage rapidly triggers ILC precursors to differentiate into mature ILCs following cytokine‐driven expansion.^21^, ^22^ ILCs do not express antigen‐specific receptors, and cytokines are considered as powerful activators of ILCs. ILCs can directly respond to epithelial cytokines in the absence of T cells and even respond to ‘risk signal’ faster than T cells.^23^, ^24^, ^25^ Furthermore, ILC2s activated by IL‐33 may produce 10 times more type 2 cytokines than Th2 cells do.^26^

ILCs are involved in the first‐line immune defence by secreting a range of effect cytokines and interacting with other cells. Some evidence shows that the inflammatory response induced by epithelial cytokines is mediated by ILCs but not T cells, B cells or NK cells.^27^ Besides, IL‐25 can induce ILC2s to produce IL‐5 and IL‐13 even in RAG‐deficient mice.^28^ Intriguingly, there are a series of IL‐10‐secreting ILCs, generated in severe inflammatory environment, which in turn alleviate inflammation in different ways.^29^, ^30^, ^31^ It is revealed that the interaction between inflammatory factors and IL‐10‐secreting ILCs may initiate tissue damage repair, which may constitute a negative feedback system to guard the mucosal immune system and the homeostasis of mucosae.

The interaction between cytokines and ILCs is intricate and complex. The differentiation and function of ILCs can also be regulated by cytokines in local microenvironment.^32^, ^33^, ^34^ In the complex mucosal environment, ILCs are heterogeneous and highly plastic. The plasticity of ILCs benefits the adaptability to various inflammatory conditions. For example, lung tissue‐specific ILCregs are converted from ILC2s by epithelial‐derived retinoic acid under the induction of intense inflammatory signals in local tissues.^30^ What's more, chronic inflammation can also induce the conversion of ILCs among different subtypes in mucosal diseases. For example, ILC2s can be converted to ILC1s under the stimulation of IL‐β and IL‐12 in COPD, and the ratio of ILC1s to ILC2s is positively correlated to the severity of COPD.^35^, ^36^ ILC1s increased while IL‐22^+^ ILC3s decreased in Crohn's patients, leading to the exacerbation of chronic inflammation.^37^, ^38^ Furthermore, ILC3s can be converted to ILC1s in the inflamed intestines by the stimulation of IL‐12, which is highly associated with the development of IBD.^39^, ^40^ Therefore, ILCs are of highly plasticity, which may provide new ideas for the pathogenesis of mucosal diseases.

To sum up, ILCs are abundant in mucosal barrier, close to the damaged site, and can respond to inflammatory signal sensitively. Particularly, ILCs are of typical plasticity, which indicates that ILCs may function as a double‐edged sword in mucosal barrier. The various characteristics of ILCs indicate that they have great potential in mucosal diseases. We will elucidate how ILCs regulate the immune responses and maintain the homeostasis of mucosal barriers involving lungs and intestines.

## 4 ILCS IN THE LUNGS

**FIGURE 1:**
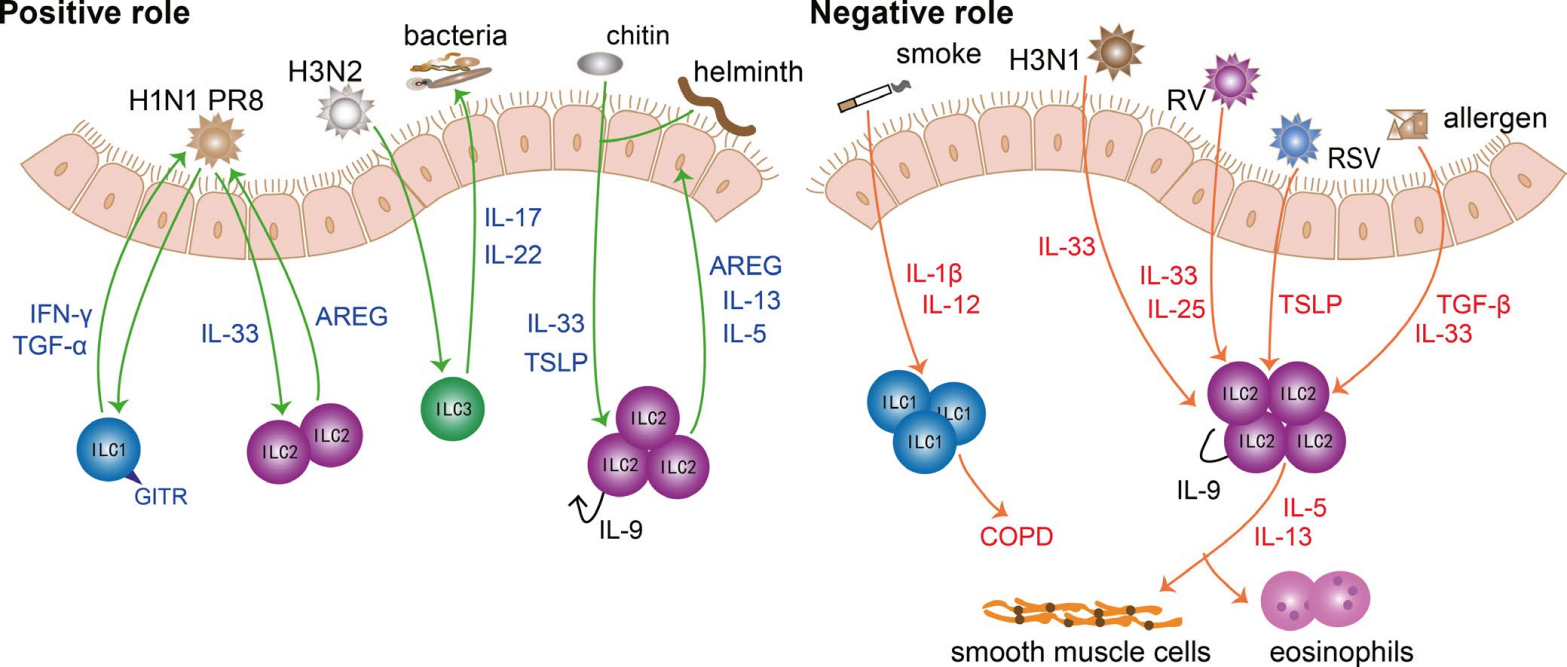
ILCs in the lungs. Different ILC subtypes play distinct roles in the lung mucosae. On the one hand, ILCs protect against various infections. On the other hand, ILCs exacerbate airway inflammation. ILC1s resist viral invasion by secreting IFN‐γ and TGF‐α. ILC2s can also contribute to helminth expulsion and tissue repair after viral invasion by amphiregulin, IL‐5 and IL‐13. However, under the stimulation of various viruses and allergen, ILC2s secret a large amount of IL‐5 and IL‐13, which induce eosinophilia infiltration, mucus hyperplasia and smooth muscle cell contraction. In addition, ILC3s effectively resist bacterial invasion and prevent lung epithelia from secondary bacterial infection after viral attack

As a tissue directly contacting the external environment, lung mucosae need to cope with airborne dust, pollen, ozone, microbes, etc. Submucosal ILCs are essential for the integrity and homeostasis of the lung mucosal barrier. Upon sensing the ‘risk signal’ from damaged mucosal epithelia, ILCs will proliferate, activate and release various cytokines to respond to harmful environmental components promptly and appropriately. Non‐recirculating ILCs in the lungs constitute a complex network, and ILCs clear irritants and promote tissue repair when viral infection and parasites attack. However, over recruitments and dysfunction of ILCs in the lungs also lead to the aggravation of chronic pulmonary diseases. Thus, we will elaborate on the function of ILCs in two aspects: the positive and negative effects of the ILCs in lung mucosae (Figure 1).

ILCs are indispensable to prevent lung from respiratory infection and maintain the pulmonary homeostasis. ILCs contribute to the defence against viral invasion, preventing secondary bacterial infection and expulsing parasites. During H1N1 PR8 infection, GITR‐expressing ILC1s contribute to preventing viral invasion through interferon (IFN)‐γ and tumour necrosis factor (TNF)‐α.^41^ Moreover, IL‐22‐producing ILC3s can help clear influenza viruses such as H3N2, alleviate inflammation and repair pulmonary epithelial integrity, which in turn prevents secondary bacterial infection.^42^, ^43^, ^44^ Strikingly, IL‐22^+^ ILC3s alleviate inflammation in allergic airway disease.^45^ Under the stimulation of chitin and helminth, IL‐9^+^‐autocrine ILC2s facilitate worm expulsion by IL‐5 and IL‐13.^46^ Of note, according to genome‐wide transcriptional profiling, lung ILCs express a series of genes related to wound healing and tissue repair, including extracellular matrix proteins decorin, asporin and dermatopontin as well as epidermal growth factor family members such as amphiregulin (AREG).^47^ ILC2‐derived AREG contributes to epithelial defence, tissue repair and restore lung function effectively.^48^, ^49^ Therefore, ILCs can not only clear irritants, but also repair damaged tissues.

It is true that ILCs play an important role in resisting invasion and facilitating tissue repair, while dysregulated ILCs also take the blame for causing disease. At present, ILCs have been confirmed to be involved in lung diseases including COPD and asthma. It has been shown that a higher proportion of ILC1s can cause exacerbations of COPD.^35^ This can be explained by the potential to transform from ILC2s to ILC1s with the help of IL‐1β and IL‐12 after exposure to cigarette smoke.^50^ Furthermore, given that IL‐17A is a detrimental factor to COPD, IL‐17‐secreting ILC3s may also participate in the pathology of COPD.^51^ The exacerbation of COPD is associated with ILC1s, while the lung functionality of asthma patients is closely related to ILC2s.^52^ The frequency of ILC2s is increased in allergic asthma patients, which are sufficient to initiate asthma.^53^, ^54^, ^55^, ^56^ After activated by IL‐25, IL‐33 and thymic stromal lymphopoietin (TSLP), ILC2s proliferate and produce amounts of IL‐5, IL‐9 and IL‐13, which in turn enhance airway inflammation, airway remodelling and airway hyperresponsiveness (AHR).^26^, ^57^, ^58^, ^59^ Especially, activated ILC2s are correlated with severe asthma and recruitment of eosinophils.^57^, ^60^, ^61^ Recent research has found that the role of TSLP in steroid‐resistant asthma is correlated with airway ILC2s.^62^, ^63^ Tezepelumab (an inhibitor of TSLP) could alleviate uncontrolled asthma, which may take effect by inhibiting TSLP‐driven ILC2s.^53^, ^64^, ^65^ What's more, viral infection can trigger acute exacerbation of asthma. After infection with H3N1 influenza virus, rhinovirus or respiratory syncytial virus (RSV), elevated IL‐25, IL‐33 and TSLP enhance the efficacy of ILC2s significantly, which in turn aggravate airway inflammation, mucus secretion and smooth muscle contraction dramatically.^66^, ^67^, ^68^, ^69^ In addition to familiar IL‐25, IL‐33 and TSLP, TGF‐β can also act as a robust activator of ILC2s, which boost ILC2‐mediated inflammatory response dramatically under the stimulation of viruses and allergens.^70^ In addition, IL‐17‐producing ILC3s promote AHR in obesity‐associated asthma.^71^, ^72^ Except for COPD and asthma, several studies have revealed that ILC2s are involved in the pathogenesis of chronic rhinosinusitis with nasal polyps and idiopathic pulmonary fibrosis.^73^, ^74^

There is no doubt ILC‐regulated mucosal immunity is of great significance to lung defence and chronic diseases. A proper ILC‐mediated defence is very beneficial to the homeostasis of lung. However, when lung mucosae are severely damaged or in a state of long‐term damage, ILCs will lead to the development of chronic pulmonary diseases. Notably, different subtypes of ILCs have different effects on the outcome of lung disease. How ILCs function in different environments and the role of ILCs plasticity in chronic diseases still need further study. Particularly, the investigation about the activation and function of ILC3s in lung mucosae is relatively rare. Besides, infections with extracellular bacteria and fungi are lack of concern in ILC‐regulated lung immunity. In‐depth studies on resident ILCs in the lungs are expected to provide new perspective to the treatment of these pulmonary diseases.

## 5 ILCS IN THE INTESTINES

**FIGURE 2:**
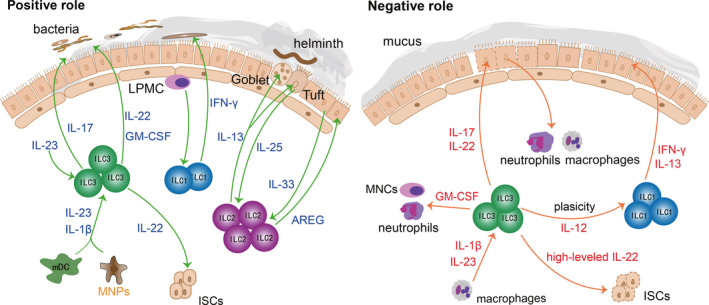
ILCs in the intestines. Different ILC subtypes play distinct roles in the intestine mucosae. ILC1s block intracellular bacterial infections by secreting IFN‐γ. ILC2s prevent helminth infection by goblet cells and tuft cells. In addition, ILC2s facilitate intestinal mucosae repair by AREG. On the one hand, ILC3s confer protection against extracellular bacterial invasion by secreting IL‐17, IL‐22 and GM‐CSF. On the other hand, hyperactivated ILC3s play a detrimental role in IBD and mucosal repair by promoting inflammatory cell infiltration and impairing ISC‐driven epithelial renewal

The intestines must process a large amount of food every day and maintain the balance of intestinal flora, so the external stimuli on the intestinal mucosal barrier are more complicated. As mentioned above, lineage‐specified progenitors of ILC3s can be detected in intestinal lamina propria.^16^ This means the intestines become a factory for producing ILC3s, and it is shown that ILC3s are the most abundant ILCs in the intestines.^75^ Moreover, intestinal flora plays an important role in maintaining the homeostasis of the intestinal tract. Due to these physiological characteristics of the intestines, there are a large number of relevant studies on the positive role of ILCs in the intestinal tract during extracellular bacterial infection and parasite invasion. In addition, the negative role of ILCs is verified by the engagements of ILCs in the pathogenesis of IBD, colitis and graft‐versus‐host disease (GVHD).^76^ Overall, ILCs are of great significance in intestinal mucosal disease, and we will also elaborate on the protective and destructive roles of ILCs in the intestines, respectively (Figure 2).

ILCs can not only protect against bacterial and parasite infections, but also promote intestinal cell proliferation and mucus secretion, which in turn reinforce the mucosal barrier. ILCs are very important for alleviating intestinal inflammation and maintaining intestinal homeostasis. ILC3s are activated by IL‐23 and IL‐1β of myeloid dendritic cells (mDC) and mononuclear phagocytes (MNPs), and then ILC3s secrete abundant IL‐22 and granulocyte macrophage colony‐stimulating factor (GM‐CSF), which in turn alleviate the symptoms of colitis patients effectively.^77^, ^78^ Besides, IL‐17‐secreting ILC3s can also recruit neutrophils to produce reactive oxygen species and α‐defensin, contributing to the resolution of inflammation in colitis patients.^76^ ILC1s are predominantly distributed within the intestinal lamina propria and participate in resisting bacteria by secreting IFN‐γ.^38^ After exposure to pathogenic bacteria, lamina propria mononuclear cells (LPMC) activate ILC1s to release IFN‐γ, which in turn resist bacterial invasion.^79^ Besides, IL‐22‐secreting ILC3s promote the secretion of antimicrobial peptides, lipocalin and mucus to prevent the invasion of bacteria.^80^ Furthermore, studies have shown that IL‐22‐secreting ILC3s can prevent the invasion of C.rodentium deep into the crypt of the colon.^81^, ^82^ In addition to fighting against bacterial infections, the intestines can also expel parasites through ILCs effectively. Tuft cells, main source of IL‐25, can sense helminth invasion sensitively and activate ILC2s promptly. Then, IL‐13‐secreting ILC2s expel helminth effectively.^83^, ^84^ Meanwhile, ILC2‐derived IL‐13 can promote the proliferation and differentiation of mucosal tuft cells and goblet cells, to reinforce the mucosal barrier and prevent secondary infection.^85^, ^86^ Intriguingly, IL‐22‐secreting ILC3s are also critical to the integrity and balance of mucosal barrier during GVHD by facilitate intestinal stem cells (ISCs) fitness.^77^, ^87^, ^88^ It has been proven that crypt cells and epithelial cells will be vulnerable after deletion of IL‐22, leading to the aggravation of gastrointestinal GVHD.^89^, ^90^ What's more, IL‐33‐activated ILC2s further upregulate the mucin production and promote the recovery of mucosal barrier by AREG.^91^

A growing number of studies have shown that excessive recruitment of cytokines and inflammatory cytokines would reverse the protective effect of ILCs on the intestinal mucosal barrier. As mentioned above, when ILC3s are activated by mDC and MNPs, ILC3s can alleviate the symptoms of colitis patients.^78^ However, it has also been reported that NKp46^+^ ILC3s could also induce inflammation in the colitis model.^92^ This can be explained by the fact that ILC3s‐derived GM‐CSF can recruit neutrophils and mononuclear cells (MNCs), which in turn destroy the intestinal tissues and further aggravate inflammation.^93^ Although modest ILC3‐derived IL‐22 can protect ISCs, redundant IL‐22 may suppress the survival of ISCs, inhibiting mucosal repair.^94^, ^95^ IBD is a typical intestinal mucosal disease. ILC3s are closely related to the prevalence of IBD. ILC3s are activated by IL‐1β and IL‐23 of macrophages. The activated ILC3s secreted a great amount of IL‐22 and IL‐17 which would accelerate the pathological process of IBD.^96^ Besides, excessive IL‐17 over‐activates neutrophils and macrophages, breaking down interepithelial adhesion molecules and destructing the intestinal mucosal barrier.^97^ ILC3s are dominant in the intestines, while ILC3s are skewed towards ILC1s that produce IFN‐γ and IL‐13 in IBD patients.^38^, ^40^ It is verified that ILC1‐derived IFN‐γ damages tight junction proteins and increases the permeability of the intestinal mucosal barrier, thereby causing microbial translocation.^98^

The interaction between ILCs and other inflammatory cells in the intestinal mucosa and the conversion of ILCs play important roles in intestinal inflammatory diseases such as colitis and IBD. However, the roles of ILCs in intestinal inflammation are controversial. ILC3s even show two completely opposite effects in colitis patients.^78^, ^92^ We speculate that inflammatory factors and immune cells in different microenvironments have a great influence on the functionality of ILCs. In‐depth exploration of the proliferation, activation and differentiation of ILCs in intestinal bacterial infections and inflammatory responses is of great significance to the maintenance of intestinal homeostasis and the treatment of intestinal diseases.

## 6 FUTURE PERSPECTIVES

ILCs actively fight against bacteria, fungi, parasites, viruses and other pathogens and play an indispensable role in maintaining mucosal homeostasis and regulating the mucosal inflammation. In recent years, there has been a growing appreciation for the role of ILCs in mucosal diseases, ILC disorders will damage the integrity of the mucosae and exacerbate the inflammation of mucosae, leading to disease deterioration. In this review, we have summarized the recent studies on the involvement of ILCs not only in health but also in diseases.

ILCs are indispensable mediators in the regulation of mucosal immunity. The expansion, activation and differentiation of ILCs are driven by cytokines in mucosal environment. The mucosal microenvironment can be a vital determinant to the activation and differentiation of ILCs, while the functions of ILCs have a great influence on mucosae. The interaction between mucosae and ILCs is complex and intricate. ILCs are competent to serve as innate counterparts of T cells to some extent. Moreover, ILCs can promote T‐cell activation and cytokine secretion.^87^, ^99^, ^100^, ^101^, ^102^ ILCs and T cells constitute an entire and intact defence system against pathogen invasion and/or tissue damage.

ILCs are heterogeneous, and ILCs are regarded as highly plastic cells that can switch their phenotype depending on the local microenvironment. Intriguingly, the conversion of ILCs has been discovered in mucosal diseases, especially chronic diseases such as COPD, Crohn's disease and IBD.^35^, ^36^, ^37^, ^38^ This may sharpen new ideas on the pathogenesis of pulmonary and intestinal chronic diseases. Whether the conversion of ILCs in chronic diseases could serve as a signal for clinical diagnosis still needs further study. In addition, the fundamental reason why the same subtype of ILCs causes completely different outcomes in the same disease model remains to be deeply investigated.

ILCs should keep a subtle balance to achieve mucosal immune defence and avoid additional tissue injury. Excessive cytokines will cause hyperactivation of ILCs, and a large number of activated ILCs may cause chronic inflammation and tissue remodelling during the long‐term inflammatory stimulation and tissue repair. An increasing number of monoclonal antibodies (targeting IL‐33, TSLP, IL‐25, etc.) have been utilized in clinical trials.^13^, ^52^, ^103^ A better understanding of how ILCs are influenced by cytokines will promote our understanding of therapeutic targets. The interaction between ILCs and cytokines will also provide a novel insight into the mechanisms of cytokine‐targeting drugs.

In summary, ILCs are of great significance to the immune response and mucosal homeostasis. Due to the intricate ILC category, single‐cell analysis of ILCs is required to better understand the phenotype, function and development of ILCs. Given the overlapping functions of T cells and ILCs, Rag^−/−^ mice are used as ideal animal models to clarify the relative contribution of ILCs and the ILC‐T cross‐regulation. Furthermore, the regulation of ILCs in the mucosal barrier may provide a new perspective for feasible therapeutic targets in the future.

## CONFLICT OF INTEREST

The authors declare no conflict of interest.

## AUTHOR CONTRIBUTIONS

**Zhen Duan:** Conceptualization (equal); Data curation (equal); Formal analysis (equal); Investigation (equal); Resources (equal); Writing‐original draft (equal); Writing‐review & editing (equal). **Mandie Liu:** Data curation (equal); Resources (equal); Writing‐original draft (equal); Writing‐review & editing (equal). **Lin Yuan:** Resources (equal). **Xizi Du:** Resources (equal). **Mengping Wu:** Resources (equal). **Yu Yang:** Resources (equal). **Leyuan Wang:** Resources (equal). **Kai Zhou:** Resources (equal). **Ming Yang:** Resources (equal); Supervision (equal). **Yizhou Zou:** Resources (equal); Supervision (equal). **Yang Xiang:** Funding acquisition (equal); Supervision (equal). **Xiangping Qu:** Funding acquisition (equal); Supervision (equal). **Huijun Liu:** Project administration (equal); Supervision (equal). **Xiaoqun Qin:** Funding acquisition (equal); Supervision (equal). **Chi Liu:** Funding acquisition (equal); Supervision (equal); Validation (equal); Writing‐review & editing (equal).

## Data Availability

Data sharing not applicable to this article as no datasets were generated or analysed during the current study.
